# The Molecular and Structural Basis of *O*-methylation Reaction in Coumarin Biosynthesis in *Peucedanum praeruptorum* Dunn

**DOI:** 10.3390/ijms20071533

**Published:** 2019-03-27

**Authors:** Yucheng Zhao, Nana Wang, Ziwei Sui, Chuanlong Huang, Zhixiong Zeng, Lingyi Kong

**Affiliations:** 1 Key Laboratory of Bioactive Natural Product Research and State Key Laboratory of Natural Medicines, School of Traditional Chinese Pharmacy, China Pharmaceutical University, Nanjing 210009, China; zhaoyucheng1986@126.com (Y.Z.); szwcpu@163.com (Z.S.); hchuanlong@163.com (C.H.); 2 National Key Laboratory of Crop Genetic Improvement, Huazhong Agricultural University, Wuhan 430070, China; wangnana@webmail.hzau.edu.cn

**Keywords:** coumarins, caffeic acid *O*-methyltransferase, bergaptol *O*-methyltransferase, *O*-methylation, evolution

## Abstract

Methoxylated coumarins represent a large proportion of officinal value coumarins while only one enzyme specific to bergaptol *O*-methylation (BMT) has been identified to date. The multiple types of methoxylated coumarins indicate that at least one unknown enzyme participates in the *O*-methylation of other hydroxylated coumarins and remains to be identified. Combined transcriptome and metabonomics analysis revealed that an enzyme similar to caffeic acid *O*-methyltransferase (COMT-S, S is short for similar) was involved in catalyzing all the hydroxylated coumarins in *Peucedanum praeruptorum*. However, the precise molecular mechanism of its substrate heterozygosis remains unsolved. Pursuing this question, we determined the crystal structure of COMT-S to clarify its substrate preference. The result revealed that Asn132, Asp271, and Asn325 govern the substrate heterozygosis of COMT-S. A single mutation, such as N132A, determines the catalytic selectivity of hydroxyl groups in esculetin and also causes production differences in bergapten. Evolution-based analysis indicated that BMT was only recently derived as a paralogue of caffeic acid *O*-methyltransferase (COMT) via gene duplication, occurring before the Apiaceae family divergence between 37 and 100 mya. The present study identified the previously unknown *O*-methylation steps in coumarin biosynthesis. The crystallographic and mutational studies provided a deeper understanding of the substrate preference, which can be used for producing specific *O*-methylation coumarins. Moreover, the evolutionary relationship between BMT and COMT-S was clarified to facilitate understanding of evolutionary events in the Apiaceae family.

## 1. Introduction

Methoxylated coumarins represent a large proportion of officinal value coumarins that are widely used in anti-cancer, anti-inflammatory, and anti-oxidant applications [1,2,3]. Hence, effort to secure a supply of this type of compound from medicinal plants has been going on for thousands of years. However, this presents a considerable challenge to facilities that acquire these compounds by the means of solvent extraction. Metabolic engineering is a promising method for addressing these problems [4,5,6]; however, major hurdles such as low yield and an unclear biosynthetic pathway remain to be resolved before scale-up can be achieved for most coumarins [7]. In addition, there has been little structural information discovered about the proteins involved in coumarin biosynthesis [8].

*O*-methylation is one of the most important reactions in coumarin biosynthesis; it creates a series of structurally modified compounds (e.g., xanthotoxin, bergapten, scopoletin, and isoscopoletin, Figure S1) [9,10,11]. However, little is known about the methylation mechanism of coumarin derivatives [9,12,13]. Moreover, despite the first successful cloning of coumarin-specific bergaptol *O*-methylation (BMT) in 2004, there is currently no information regarding how esculetin and other hydroxylated coumarins are *O*-methoxylated (Figure S1) [11,14]. The strict substrate specificity of BMT indicates that at least one unknown enzyme participating in the *O*-methylation of other hydroxylated coumarins remains to be identified [9,14].

*P. praeruptorum* is a well-known agent used in traditional Chinese medicine and has been used for more than 1500 years to treat colds, coughs with abundant phlegm, and chest congestion [15,16]. It is the main source of coumarins in China and also is listed in the current Pharmacopeia of China. As it contains almost all types of coumarins (prenylated coumarins, linear/angular furanocoumarins and pyranocoumarins, etc.), it is suitable for investigating their biosynthetic pathways [11,17,18]. To identify the unknown enzyme participating in the *O*-methylation reaction of coumarins, the transcriptome of *P. praeruptorum* in different tissues, and methyl jasmonate (MeJA) treatment were constructed for the first time in this study. Through combined bioinformatics analysis with biochemical identification, an enzyme similar to caffeic acid *O*-methyltransferase (COMT-S) was identified as being responsible for the identified *O*-methylation reaction in coumarin biosynthesis. Next, this enzyme’s physiological and biochemical characteristics were investigated. However, some issues still need to be clarified: (a) The substrate heterozygosis of COMT-S (b) in the circumstances that COMT-S could methylate every coumarins, as well as when and how bergaptol *O*-methylation (BMT) evolved.

Herein, we determined the crystal structure of COMT-S. Structural analysis and mutation studies provided insight into its precise catalytic mechanism in the coumarin-specific *O*-methylation reaction. In addition, the molecular mechanism underlying substrate heterozygosis was also clarified. Finally, an evolutionary analysis was conducted regarding the evolutionary history of the Viridiplantae to identify when and how BMT evolved [19]. The work described here is the first report focused on the biosynthesis of xanthotoxin, isoscopoletin, and scopoletin, the unknown step in coumarin biosynthesis [9,11]. The study also provides a deep understanding of the substrate preferences and the catalytic mechanism accompanying COMT-S mediated *O*-methylation of coumarins. In addition, the evolutionary relationship of BMT and COMT-S are also investigated.

## 2. Results and Discussion

### 2.1. Candidate Gene Mining with Transcriptome Sequencing and Phytochemical Analysis

**Figure 1:**
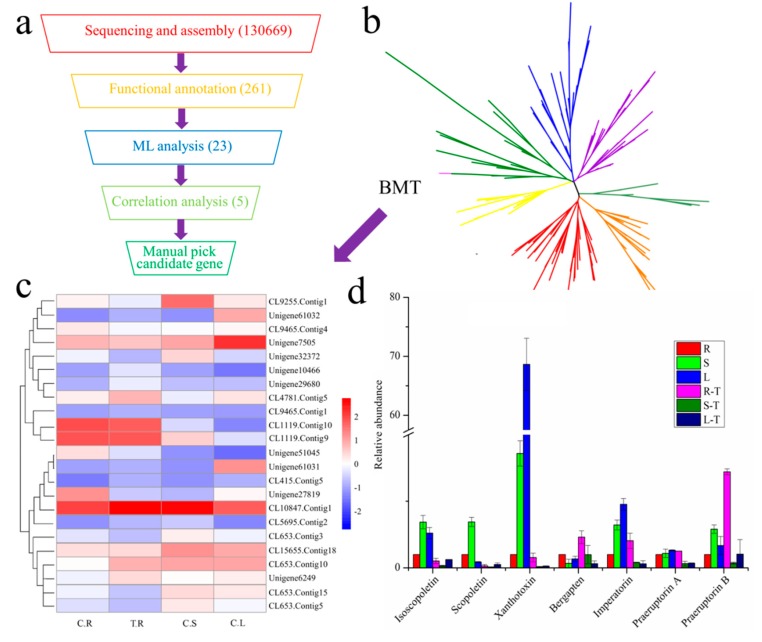
Candidate genes mining using transcriptome sequencing and phytochemical analysis. (**a**) The overall experimental design of candidate genes mining used in this study. (**b**) Phylogenetic tree of all the 151 non-redundancy OMTs constructed by maximum-likelihood. Different OMTs are marked with different colors. Bergaptol *O*-methylation gene (corresponding to CL1119.contig10 in the present transcriptome dataset) was marked with pink to locate the candidate genes. (**c**) Expression heatmap of candidate genes and (**d**) relative abundance of main coumarins and all the *O*-methoxylated coumarins in *P. praeruptorum*. R, S, L, C, T represents root, stem, leaf, control (without treatment) and treatment with methyl jasmonate (MeJA), respectively. The abundance of each compound in root without treatment was used as reference and set as 1 for normalization. The data represent the means ± SD of 3 replicates and the folds to the control.

Despite the biochemical characterization of enzyme activities involved in *O*-methylation of coumarins 30 years ago, only the *bmt* gene has been cloned in recent years [9,14,20]. To authenticate the candidate missing genes involved in the *O*-methylation of coumarins, we employed *P. praeruptorum* as a research material, as it contains almost all types of coumarins [11,17,18]. The experiment was originally conducted to identify candidate genes involved in the *O*-methylation of coumarins. Hence, the transcriptome of *P. praeruptorum* for various tissues and root tissue treated with methyl jasmonate (MeJA) was constructed with the bioproject accession number PRJNA359149 in the National Center for Biotechnology Information (NCBI). This produced about 189 million clean reads and 130,669 unigenes/transcripts with mean length of 668 nt and N50 of 1271 nt after de novo assembly (Table S1 and Figure S2). Then, the transcripts and unigenes were functionally annotated with the public database to predict and classify their possible functions (Figure S3). Ultimately, a total of 261 transcripts, or unigenes, were functionally categorized as OMTs (*O*-methyltransferases) (Figure 1a, Table S2). Redundancy of OMTs was removed to construct a phylogenetic tree using maximum-likelihood to distinguish the different OMTs (Figure 1b, Figure S4). Considering the similarity of chemical structures in coumarins, the *bmt* gene (corresponding to CL1119.Contig10 in the present transcriptome dataset) was used as a reference to locate the candidate genes [9]. As indicated in Figure 1b,c, 23 OMTs had a higher similarity with *bmt* and may serve as coumarin-specific OMTs. Considering the consistency of *bmt* expression and differences in bergapten content in different tissues and treatments, we deem the expression mode of candidate OMTs to be similar to that for scopoletin, isoscopoletin, and xanthotoxin content (Figure 1d). Hence, five genes, CL9255.Contig1, Unigene32372, CL653.Contig3, CL653.Contig5, and CL653.Contig15, were selected as candidate OMTs. Subsequently, we compared the functional annotation results of the five genes with BMT. Ultimately, CL9255.Contig1 had the most similar function to BMT, despite variance in Nr, Nt, Swissprot-annotation, and molecular function.

### 2.2. Functional Characterization of COMT-S

**Figure 2:**
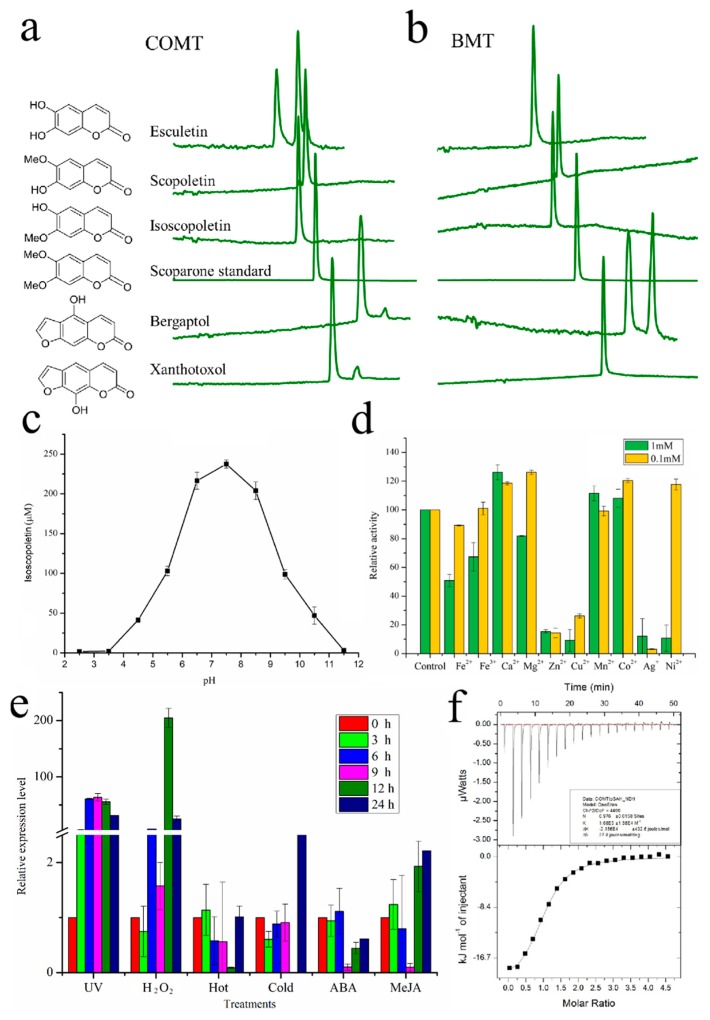
Functional characterization of COMT-S. HPLC analysis of the enzymatic reaction products generated by recombinant COMT-S (**a**) and PpBMT (**b**). Different hydroxylated coumarins were used as substrates and their chemical structures were marked on the left. COMT-S could accept esculetin, bergaptol, and xanthotoxl as substrates, while BMT could only catalyze bergaptol. Effect of pH (**c**) and metal ions (**d**) on the catalytic activity of recombinant COMT-S. The pH tolerance was conducted in a pH range 2.5 to 11.5. The effects of different metal ions (Fe^2+^, Fe^3+^, Ca^2+^, Mg^2+^, Zn^2+^, Cu^2+^, Mn^2+^, Co^2+^, Ag^+^, and Ni^2+^) in different concentrations (both in 1 and 0.1 mM) on COMT-S were displayed in relative activity. The activity of COMT-S without ions treatment was set as 100%. (**e**) Expression profiles of COMT-S. Different treatments (UV, H_2_O_2_, Hot, Cold, ABA, MeJA) were listed in the X axis and their relative expression level were shown as fold changes to the expression level of COMT-S without treatment (0 h). Different colors indicate different treatment time. All the data represents means ± SD of three replicates. (**f**) ITC (Isothermal Titration Calorimetry) analysis of COMT-S.

We cloned the full-length coding position site (CDS) of CL9255.Contig1 from *P. praeruptorum* cDNA to analyze biochemical activity. The protein, identical to caffeic acid 3-*O*-methyltransferase in *Angelica sinensis*, *Anthriscus sylvestris*, and *Daucus carota*, was named COMT-S for the purpose of this work. The protein was expressed and purified to determine enzymatic activity with different coumarins. Surprisingly, the candidate protein displayed significant activity with esculetin, bergaptol and xanthotoxol, indicating that it has a broad substrate preference. Interestingly, scopoletin and isoscopoletin, the methylated esculetin in 6-OH and 7-OH, could not be dimethylated by COMT-S (Figure 2a). Considering that scoparone was not identified in *P. praeruptorum*, the results above can be interpreted readily [17]. However, the strict substrate specificity of BMT makes it functional only for bergaptol (Figure 2b) [9]. Biochemical characteristics indicated that COMT-S had an optimal pH of 7.5 and could be inhibited by Zn^2+^ and Ag^+^ (Figure 2c,d). We previously proved that BMT exhibited a tissue-specific expression. However, COMT-S had similar expression in all tissues except stem tissues, which was consistent with the scopoletin/isoscopoletin content in *P. praeruptorum* (Figure 1d) [9]. In addition, COMT-S had little susceptibility to MeJA, hormones, and environmental factors (cold and hot) but was strikingly affected by UV and H_2_O_2_ (Figure 2e). This indicated that COMT-S and BMT might participate in differing aspects of biological activity in *P. praeruptorum*. For instance, bergapten, the *O*-methoxylated psoralen, is the most relevant natural furanocoumarin in treating psoriasis and vitiligo, while scopoletin, a phytoalexin, facilitates resistance to microbial infection and other stresses (mechanical injury, dehydration) [21,22,23]. To survive biotic and abiotic stresses, plants have developed elaborate mechanisms; COMT therefore occurs ubiquitously in plants. In contrast, BMT was only identified in a few specific species in the Apiaceae family [9,14,24]. Through the analysis above, we can come to the conclusion that despite COMT-S explaining the *O*-methylation reaction of coumarin biosynthesis in *P. praeruptorum*, the evolution of BMT was also necessary for its special function in bergapten production [21].

### 2.3. Docking of COMT-S to Identify the Key Residues Involved in SAH/Esculetin Binding

**Figure 3:**
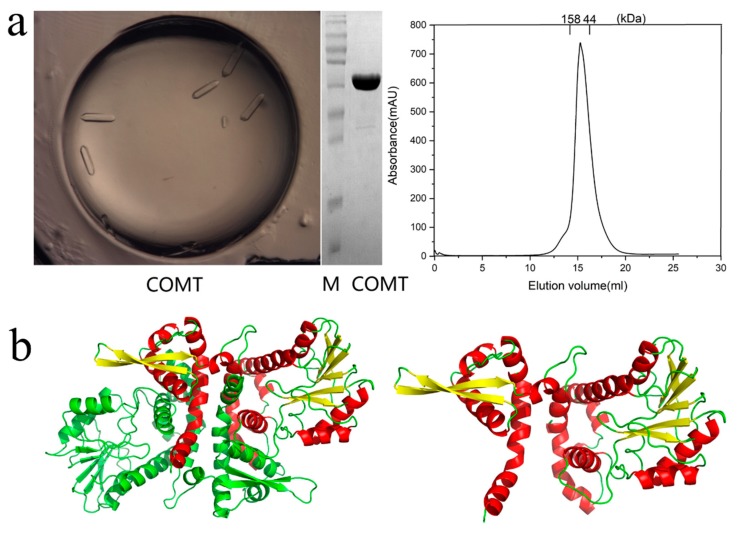
Crystallization, SDS-PAGE, and elution volume analysis of purified COMT-S protein (**a**) and its apo form crystal structure (**b**). M, the molecular mass of the ladder, indicated 100, 70, 55, 40, 35, and 25 kDa from top to bottom.

**Figure 4:**
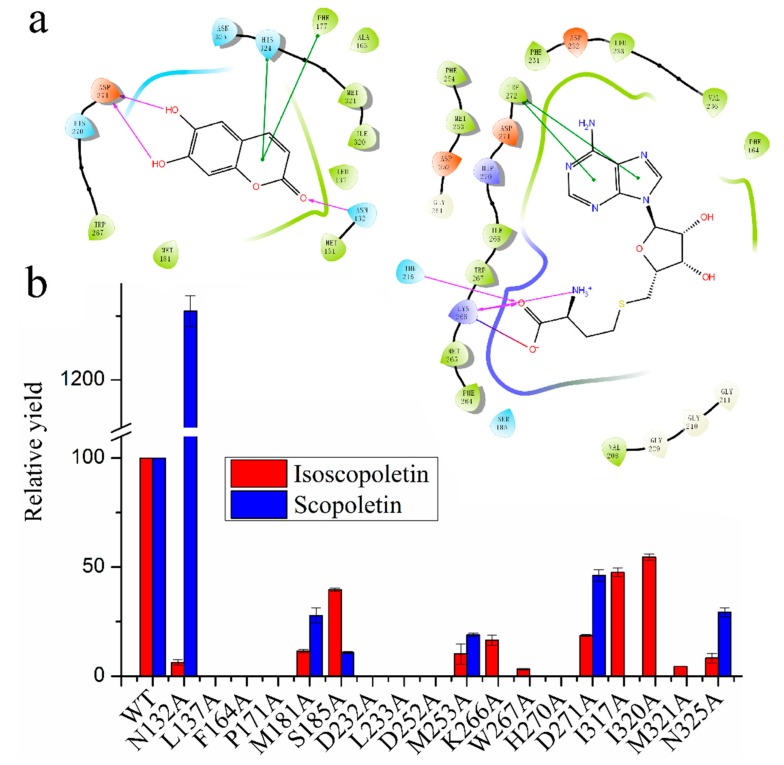
Docking of COMT-S with esculetin and *S*-adenosyl-l-homocysteine (SAH) (**a**) and the relative activity of COMT-S and its mutants toward esculetin (**b**). The activity of wild type COMT-S was calculated by its two products, isoscopoletin (red) and scopoletin (blue). Their yield was set as 100% for normalization. Analysis and reaction process is listed in the methods part. The data represents the means ± SD of three replicates.

**Figure 5:**
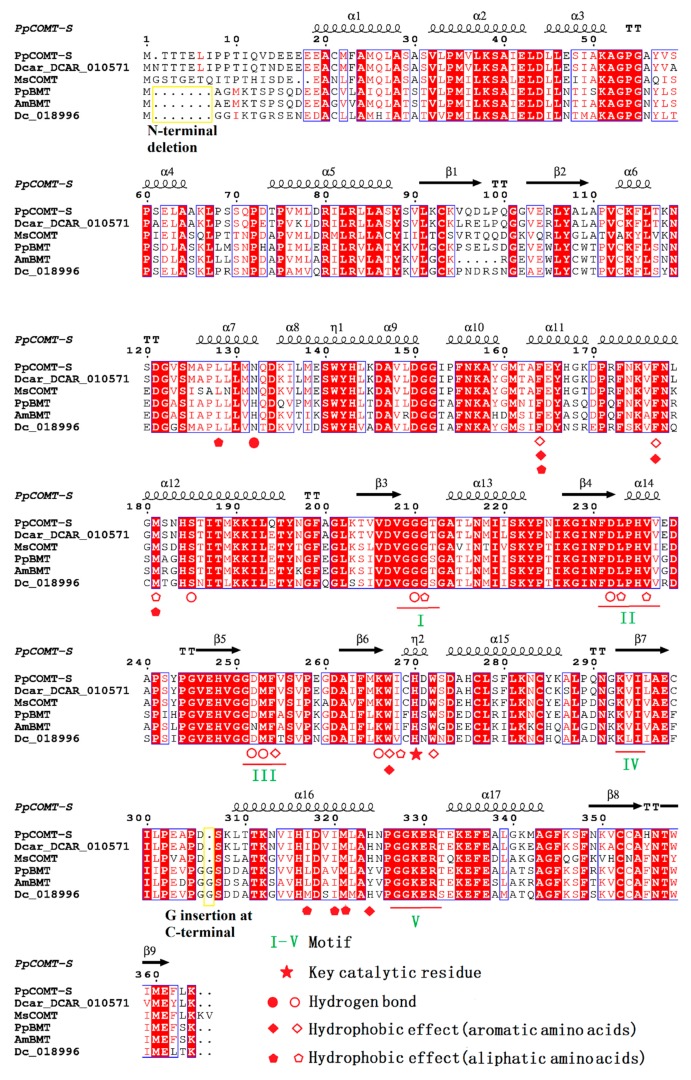
Structural and sequence alignment of representative OMTs. The accession numbers of the six representative OMTs are ANA75355.1 of PpBMT, AY443006 of AmBMT, and 010571/018996 in the *D. carota* genome. The secondary-structural elements occurring in the COMT-S structure are indicated above and the alignment with helix (α-helices) and right arrow (β-strands). Numbering of COMT-S is also labeled above with every 10th position dotted. The motifs of COMT are marked with I to IV and the key catalytic residue is indicated with a star. Residues involved in hydrogen, hydrophobic effect with aromatic amino acids, and hydrophobic effect with aliphatic amino acids are marked with a circle, quadrilateral, and pentagon, respectively. The solid and hollow of the shapes are specific for bergaptol and SAH. BMT and COMT from different species are marked with a yellow rectangular box and N-terminal deletion in BMT and G (glycine) insertion in C-terminal are marked with a vertical box.

Despite a number of reports on the catalytic mechanism of OMTs, no research has focused on the methylation mechanism of coumarin derivatives, and the substrate heterozygosis of COMT-S and substrate specificity of BMT remains unknown [9,12,13]. To investigate their difference, the crystal of COMT-S was firstly determined at a resolution of 2.53 Å (Protein Data Bank (PDB) accession number 6IWT), but we failed to obtain its ternary complex (Figure 3, Table S3). Hence, a structure-based docking study was conducted to identify the key residues involved in esculetin/S-adenosyl-L-homocysteine (SAH) binding (Figure 4, Figure 5 and Figure S5). The overall structure indicated that they share similarities in sequence and structural conservation. As indicated in Figure 4a and Figure S5, nine hydrogen bonds were formed between SAH and Ser185, Gly209, Asp232, Asp252, Met 253, Met265, and Lys266 in COMT-S to facilitate transmethylation. Hydrophobic pockets formed by amino acids 264–268, 231–236, and 251–254 participated in stabilizing the cofactor. Mutations of Ser185, Asp232, Leu233, Asp252, Met253, Lys266, and Trp267 into alanine all reduce or abolish its activity, indicating that these amino acids play an important role in SAH binding (Figure 4b). The substrate pocket is relatively spacious, which facilitates the accommodation of esculetin, bergaptol, and xanthotoxol. Esculetin is trapped in a hydrophobic pocket consisting of Leu137, Phe164, Phe173, Phe177, Met181, Trp267, and Met321 in COMT-S (Figure 4, Figure S5). Their mutation into alanine nearly abolishes their activity, indicating that the hydrophobic pocket is important for substrate binding (Figure 4b). The side chain of Asp271 forms two hydrogen bonds with 6, 7-hydroxyl of esculetin, in addition, Asn325 and Asn132 also have hydrogen bonding interactions with 6-hydroxyl and 2-carbonyl oxygen of esculetin. Asn132, Asp271, and Asn325 also oriented the substrate properly and determined the catalytic preference. Generally, the SAH binding site is relatively conserved in all the OMTs, and the substrate pocket is varied and relatively spacious which may be involved in its substrate heterozygosis.

### 2.4. Sequence Alignment and Biochemical Analysis to Identify the Key Residues Involved in Substrate Heterozygosity

**Figure 6:**
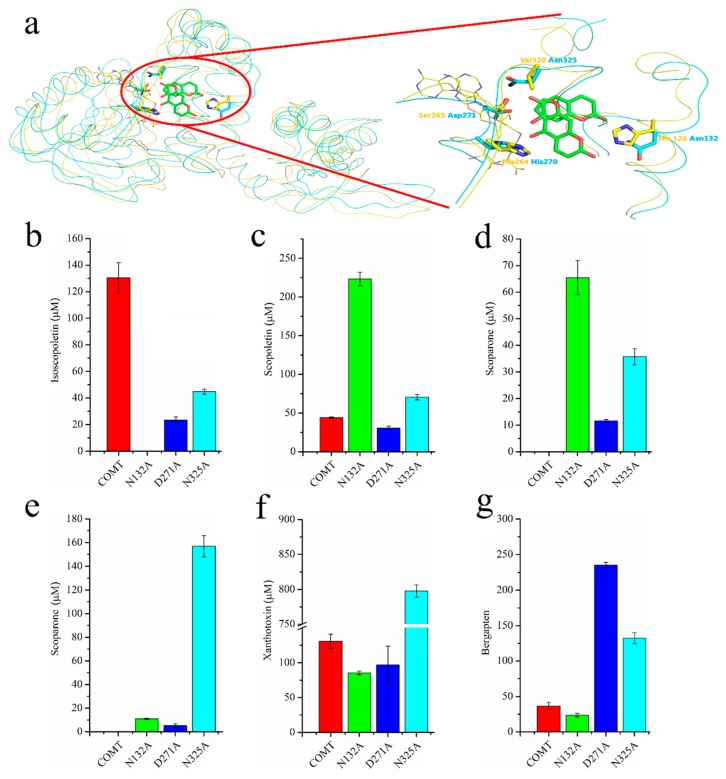
The structure comparison of PpBMT with COMT-S and their mutants towards different hydroxylated coumarins. (**a**) The structure comparison of PpBMT with COMT-S and its local enlarged form. A relatively high similarity was observed in BMT and COMT-S indicated that COMT –S could catalyze bergaptol structurally. The colors of BMT and COMT-S are yellow and blue, respectively. (**b**–**g**) COMT-S (and its mutants N132A, D271A, N325A) in catalyzing esculetin (**b**,**c**), isoscopoletin (**d**), scopoletin (**e**), xanthotoxl (**f**) and bergaptol (**g**) to produce isoscopoletin (**b**)/scopoletin (**c**), scoparone (**d**,**e**), xanthotoxin (**f**) and bergapten (**g**), respectively.

Analyzing the structure docking results and sequence alignment of PpCOMT-S to other OMTs, we found that they share a relative similarity in sequence and structure, and have a conserved pocket for *S*-adenosyl-l-methionine (SAM) binding (Figure 4, Figure 5 and Figure S5). Nevertheless, COMT-S has a broad substrate specificity, including esculetin, bergaptol, and xanthotoxol, and PpBMT exhibits strict substrate specificity to bergaptol. Structural and sequence alignment of COMT-S with BMT clearly reveals that amino acids Met131 (Val125), Leu137 (Pro131), Ala163 (Ile157), His167 (Ala161), Ile320 (Val315), and His324 (Tyr319), especially Asn132 (His126), Asp271 (Ser265), and Asn325 (Val320) govern the spatial arrangement of substrate pockets (Figure 4, Figure 5 and Figure 6a). The docked esculetin, xanthotoxol, and bergaptol have no steric hindrance with any amino acids in COMT-S, which is satisfied by the primary condition of the catalytic reaction (Figure S6). The 6,7-hydroxyl group of esculetin was pulled by Asn325 and Asp271, which is closer to the catalytic amino acid His270 and donor SAM. Mutation of the amino acids into alanine reduces their activity, indicating the importance of the hydrogen bond between Asn325, Asp271, and esculetin (Figure 6b). Another hydrogen bond between the 2-carbonyl oxygen of esculetin and the amide group of Asn132 strengthens the binding of esculetin with COMT-S, facilitating methylation of the 7-hydroxyl group of esculetin (Figure S6a). However, while catalyzing the 6-hydroxyl group of esculetin, the 2-carbonyl oxygen of esculetin is oriented toward the hydrophobic amino acid Leu127, resulting in slightly unstable interactions and leading to lower production of scopletin, which is in accordance with the enzyme activity in vitro (Figure S6b, Figure 6b,c). Interestingly, when we observed the mutation of Asn132 into alanine, only scopoletin was produced in a high yield, indicating that Asn132 determined the catalytic selectivity between the 6-hydroxyl and 7-hydroxyl groups in esculetin (Figure 6b,c). In addition, all three mutations displayed the catalytic activity to scopoletin and isoscopoletin that could not be realized in COMT-S (Figure S5, Figure 6). The amide group of Asn325 and carbonyl oxygen of Asp271 formed two hydrogen bonds with furan oxygen, and the 8-hydroxyl group of xanthotoxol, respectively. The amide group of Asn132 formed a hydrogen bond with pyran-2- ketone (Figure S6c,d). As a result, the 8 or 5-hydroxyl group was close to the catalytic amino acid His270 and SAM, insuring its catalytic activity with xanthotoxol and bergaptol (Figure S5, Figure 6f,g). Surprisingly, the enzyme activity of N325A and D271A increased towards xanthotoxol and bergaptol, respectively. The hydrogen bonding interactions between Asp271, Asn325, and Asn132 with esculetin, and Asn325, Asp271, His270, and Asn132 with bergaptol make it clear that COMT-S functions thusly with all the coumarins examined.

### 2.5. BMT Was Evolved as a Special Enzyme from COMT-S by Gene Duplication

Since COMT-S explains the *O*-methylation reaction in coumarins biosynthesis, when and how BMT evolves remains to be resolved. To investigate the evolution of BMT, we constructed the genetic evolutionary tree based on the protein sequences of 28 representative species in the Viridiplantae [19]. In particular, species with genome sequenced in Asterids were all selected for their potential close evolutionary relationship with *P. praeruptorum* (the same family as *Daucus carota*).

**Figure 7:**
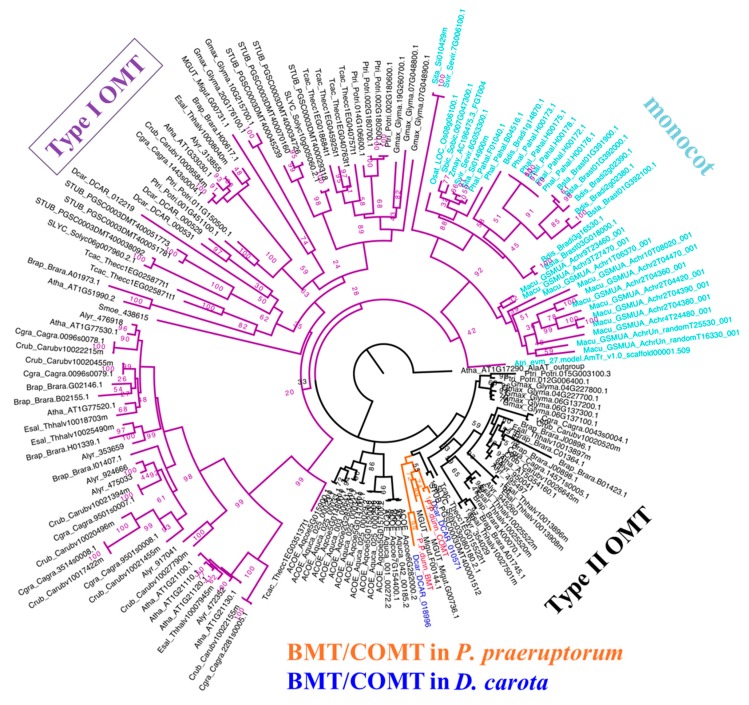
The evolution of BMT and COMT-S. There are two types of OMT in higher plants, type I OMTs (purple branch) present both in monocot (light blue leaves) and dicot (black leaves), and type II OMTs (black branch) exist only in dicot. BMT and COMT-S are both found in *P. praeruptorum* (red leaves) and *D. carota* (blue leaves), the two species of which belong to Asterids (orange branch).

As indicated in Figure 7, there were two distinct types of OMTs, with type I OMTs presented in both monocot and dicot and type II OMTs presented only in dicot. COMT-S and BMT belonging to type II OMTs indicated that they have a close genetic relationship. Interestingly, BMT only presented in *P. praeruptorum* and *D. carota*, suggesting that BMT originated as the most recent common ancestor (MRCA) of *P. praeruptorum* and *D. carota* between 37 mya and 100 mya (Figure S8a). Considering the fact that the identified BMT genes are all from the Apiaceae family, we can come to the conclusion that BMT may have evolved with the divergence of euasterid II clade [9,14,24]. Moreover, we found that the BMT and COMT-S found in *P. praeruptorum* and *D. carota* shared a similar genomic structure (both COMT-S and BMT have four exons), indicating that BMT was a recent paralogue of COMT-S, derived from gene duplication instead of different splicing forms (Figure S8b). This may be in concordance with the Dc-α and Dc-β whole-genome duplications in *D. carota* and earlier γ paleohexaploidy events shared by all eudicot [25]. Despite the absence of an available genome in *P. praeruptorum*, the estimated genome size of 1.84 Gb (Figure S9) indicated that the analogous whole-genome duplication also occurred in *P. praeruptorum*, which may have led to the emergence of BMT. In addition, BMT showed a seven-amino acid deletion at the N-terminal and a one-amino acid insertion at the C-terminal in the primary structure compared to COMT-S (Figure 5). However, there was no observed difference in their three-dimensional structure.

## 3. Materials and Methods

### 3.1. Protein Expression and Purification

The open reading frame of COMT-S (NCBI accession number MK005887) was amplified from *P. praeruptorum* according to the sequence of CL9255.Contig1 in Table S2. Then, the fragment was ligated into the expression vector pET28a for protein expression and purification. For COMT-S purification, 6× His purification label according to our previously report was used [26]. The protein was further purified via gel-filtration chromatography on a Superdex 200 column (GE Healthcare, Pittsburgh, PA, USA) equilibrated with running buffer (RB) (25 mM Tris-HCl pH 8.0, 150 mM NaCl). The purified protein was analyzed by sodium dodecyl sulfate-polyacrylamide gel electrophoresis (SDS-PAGE). Finally, the purified protein was concentrated to 20 mg/mL (Amicon, 10 kDa cutoff, Millipore, MA, USA) for crystallization and other assays and stored at −80 °C.

### 3.2. Crystallization and Structure Determination

Crystallization experiments were performed using the sitting-drop vapor diffusion method at 4 °C by mixing equal volumes (0.5 μL) of protein and reservoir solution. To obtain the SAH/ esculetin–COMT-S ternary complex, we also mixed COMT-S: SAH: esculetin in a molar ratio of 1:1.2:1.2. Eventually, we obtained the COMT–SAH complex at 0.1 M MES (2-(N-morpholine) ethylsulfonic acid) at pH 6.0 and 22% *v*/*v* PEG (polyethylene glycol) 400. The crystals were flash-frozen in liquid nitrogen and cryoprotected by adding glycerol to a final concentration of 20%. The crystals were all diffracted to 2.0 Å at the Shanghai Synchrotron Radiation Facility on beamlines BL19U1. The dataset was processed with HKL3000 [27]. Further processing was performed with programs from the CCP4 suite [28]. Data collection and structural refinement statistics are summarized in Table S3. The apo structure was solved by molecular replacement with chain A of PDB structure 1KYZ as a search model using the program PHASER, and the ternary complex structure was solved by Autosol in PHENIX [12,29]. The structure was manually and iteratively refined with PHENIX and COOT [29,30] and then deposited to the PDB database with the accession number of 6IWT. All figures representing structures were prepared with Pymol (1.7.4, http://www.pymol.org).

### 3.3. Enzymatic Activity Assays of PpBMT and COMT-S

All enzymatic activity assays were performed in triplicates according to the methods published in previous reports, but with some minor modifications [9]. For COMT-S protein activity analysis, 10 μg protein with 1 mM different coumarins and 5 mM SAM were conducted in a total volume of 200 μL 0.2 M potassium phosphate buffer (PBS, pH 7.5). The reaction was started with the addition of SAM in a temperature controlled instrument with a constant temperature of 25 °C and ended with the addition of 20 μL 20% trichloroacetic acid (TCA). In addition, the experiment was also conducted in different pH values (2.5 to 11.5) and with different ions (Fe^2+^, Fe^3+^, Ca^2+^, Mg^2+^, Zn^2+^, Cu^2+^, Mn^2+^, Co^2+^, Ag^+^, and Ni^2+^ in 1 and 0.1 mM). All reactions were maintained for 30 min. All the mutations were generated by PCR method with KOD-plus-neo (TOYOBO, Osaka, Japan). The primers used in this experiment are listed in Table S4. After PCR, the PCR products were digested with the DpnI restriction endonuclease. The mutants were then transformed into chemically competent *Escherichia coli* DH5α cells and the plasmids from positive strains were subsequently extracted for sequencing. Positive plasmids were subsequently transferred to *E. coli* BL21 (DE3) for protein expression, purification, and analyzing the enzymatic activity.

### 3.4. HPLC/Electrospray-Ionization Quadrupole Time-of-Flight Mass Spectrometry (Q-TOF MS) Analysis of Coumarin Compounds Measurement

HPLC (Agilent Technologies, Santa Clara, CA, USA, instrument type 1200) and Q-TOF MS (Agilent Technologies, Santa Clara, CA, USA, instrument type 6250) analysis were conducted according to the previous reports [9]. For sample treatment, the reaction broth was extracted with threefold of reaction broth ethyl acetate for three times, and then the combination of extract was evaporated using a nitrogen gas instrument. At last, the residue was suspended with 1 mL methanol for further analysis. For HPLC and Q-TOF MS analysis, the solvent gradient conditions A (methanol): B (H_2_O with 0.1% formic acid) (*v*/*v*) was as follows: 0 min, 40:60; 5 min, 95:5; 15 min 95:5 was used. The compound was ultimately identified using standard and MS analysis. For each compound, the content of root without treatment was used as a control and set as 1 to normalize the abundance from other tissue or treatment. Three biological repeats were conducted for each sample.

### 3.5. Q-PCR Analysis and Induction of COMT-S Expression by Elicitors

All the experiments were conducted in six-month-old *P. praeruptorum*. Different elicitors, such as MeJA, H_2_O_2_, UV, cold, and heat were added in the medium to induce the expression of COMT-S according to our previous report [9,31]. For details, MeJA (0.2 mM), ABA (0.3 mM), and H_2_O_2_ (50 mM) were used for hormones and oxidative stress treatment. Culture temperature at 4 °C and 42 °C was used for cold and heat shock treatments. For UV elicitation, the plant, which was rotated every 2 h to minimize positional effects, was irradiated using a monochromatic lamp (312 nm) set at a distance of 15 cm. Leaves of *P. praeruptorum* after treatment were harvested at 0, 3, 6, 9, 12, and 24 h after elicitation. For each treatment, plants without treatment (in a growth chamber with a long photoperiod at 25 °C, 40%–65% relative humidity, 3000 lux of light intensity and adequate water) were set as the control, except MeJA treatment, which used the equivalent amount of ethanol in deionized water as the control plants to remove the effect of the solvent. To determine the expression level of COMT-S in different tissues, roots, stems, and leaves were also collected. Total RNA was isolated with TransZol Plant reagent (TransGen Biotech, Beijing, China) according to the manufacturer’s recommendations. After reverse transcription, the cDNA samples were used for COMT-S expression analysis by Q-PCR. Q-RT-PCR was carried out in 20 µL reaction volumes containing each primer, AceQTM qPCR SYBR Green Master Mix (Vazyme, Nanjing, China) and template DNA using a LightCycler 480 instrument (Roche Molecular Biochemicals, Mannheim, Germany). The amplification protocol was as follows: Five min of denaturation at 95 °C, 40 cycles of 95 °C for 30 s, 60 °C for 30 s, followed by one cycle of 95 °C for 15 s, 60 °C for 60 s, and 95 °C for 15 s. The relative transcript levels of the COMT-S were normalized by SAND family protein with the formula 2^−ΔΔCT^ according to our previous reports [31,32]. Three biological repeats were conducted for each experiment.

### 3.6. Transcriptome Sequence, Assembly, Bioinformatics Analysis, and Docking

To find the candidate OMTs, the transcriptome of *P. praeruptorum* was firstly constructed using our previous reported methods, and the data was deposited to the NCBI with the bioproject accession number PRJNA359149 [9]. Then, assembly and functional annotation was conducted to classify the candidate OMTs, as listed in Table S2. After filtrate, 151 non-redundancy OMTs were used for phylogenetic tree construction by maximum-likelihood. Multiple sequence alignment was performed using muscle and/or ENDscript2.0 (http://endscript.ibcp.fr/ESPript/ENDscript/). For molecular docking of different coumarins to PpBMT and COMT-S, the program Molecular Operating Environment (MOE, 2013.08, Chemical Computing Group Inc., Montreal, QC, Canada) was employed by the co-author Chuanlong Huang. To obtain more precise results, induced fit docking was also employed, whereby the flexibility of amino acid residues around the substances and the distance of 5 Å were taken into account [33]. If there were no specific requirements, all parameters were set to default.

### 3.7. Isothermal Titration Calorimetry

The equilibrium dissociation constants of PpBMT/COMT–SAH interactions were determined by an MicroCal Auto-ITC200 calorimeter (MicroCal, Malvern, UK). The binding protein PpBMT/COMT (50–100 μM) and cofactor analogue SAH (500 μM) were measured in the buffer (25 mM Tris-HCl, pH 8.0, 150 mM NaCl) at 25 °C. ITC data were analyzed and fitted in Origin 7 (OriginLab, Northampton, MA, USA) to determine the site-binding model that produced a good fit for the resulting data.

### 3.8. Evolutionary Analysis of BMT and COMT-S

The protein sequences of representative species were downloaded from Phytozome [19]. Representative species were chosen according to the following three criterions: (1) Has a close evolutionary relationship with *P. praeruptorum* (*D. carota*); (2) use one species to represent each main lineage (e.g., *Micromonas pusilla* represents *M. pusilla* chlorophyte, and *Marchania polymorpha* represents the ancestral type of embryophyte); (3) using a lineage to represent monocot and dicot, respectively (e.g., Brassicaceae to represent dicot and grass to represent dicot). In the end, 28 representative species were selected for further analysis in this work. An ortholog of BMT and COMT-S were recalled from the representative species by applying BLASTP with an *E*-value threshold of 10^−5^ [34]. Orthologous protein sequences were then aligned applying MUSCLE [35]. A phylogenetic tree was constructed applying Raxml based on protein sequence using PROTGAMMAILG (general time reversible amino acid substitution model with assumption that variations in sites follow gamma distribution) [36]. Bootstrap score was obtained based on 1000 trees. AlaAT (AT1G17290) from *Arabidopsis thaliana* was used as the out-group.

### 3.9. Statistical Analysis and the Preparation of Graphs

Unless special comments, three replicates were used to obtain the data and were presented as the mean of triplicate experiments ± standard deviation (SD). Unless produced with the specific software (MEGA5, for instance), the original graphs were generated using OriginPro 8 (OriginLab Corporation, Northampton, MA, USA), Microsoft Office PowerPoint 2010, or GraphPad Prism software. When it was necessary, the graphs were merged with Adobe PhotoShop CS6 (V13.0.1.1).

## 4. Conclusions

In summary, COMT-S plays the role of multifunctional enzymes in explaining the *O*-methylation reaction in coumarin biosynthesis, especially in the missing step. This multifunctional activity was determined by Asn325, Asp271, and Asn132. However, BMT is a strict mono-functional enzyme. This kind of substrate specificity emerged from COMT-S by gene duplication, which is in concordance the whole-genome duplication of *P. praeruptorum*. The present work revealed the missing step in coumarin biosynthesis and also in the catalytic mechanism of coumarin-specific OMT. In addition, an accurate mechanism of substrate heterozygosis of COMT-S was identified, which may enhance the production of specific compounds.

## Supplementary Materials

Supplementary materials can be found at https://www.mdpi.com/1422-0067/20/7/1533/s1.

## Abbreviations

OMTs	*O*-methyltransferases
COMT	caffeic acid *O*-methyltransferase
BMT	Bergaptol *O*-methyltransferase
HPLC	High Performance Liquid Chromatography
SD	Standard Deviation
PDB	Protein Data Bank
SAM	*S*-adenosyl-l-methionine
ESC	esculetin
SAH	*S*-adenosyl-l-homocysteine
NCBI	National Center for Biotechnology Information
