# Supplementary material for: The Molecular and Structural Basis of O-methylation Reaction in Coumarin Biosynthesis in Peucedanum praeruptorum Dunn
Source: Int J Mol Sci. 2019 Mar 27;20(7):1533. doi: 10.3390/ijms20071533 (PMC6480711; doi:10.3390/ijms20071533)
Supplement: Supplementary file 1 [file ijms-20-01533-s001.pdf]

Supplementary Information

The Molecular and Structural Basis of *O*-methylation Reaction in Coumarin Biosynthesis in *Peucedanum praeruptorum* Dunn

Yucheng Zhao[1†], Nana Wang[2†], Ziwei Sui[1], Chuanlong Huang[1], Zhixiong Zeng[2*] and Lingyi Kong[1*]

Supplementary Figures

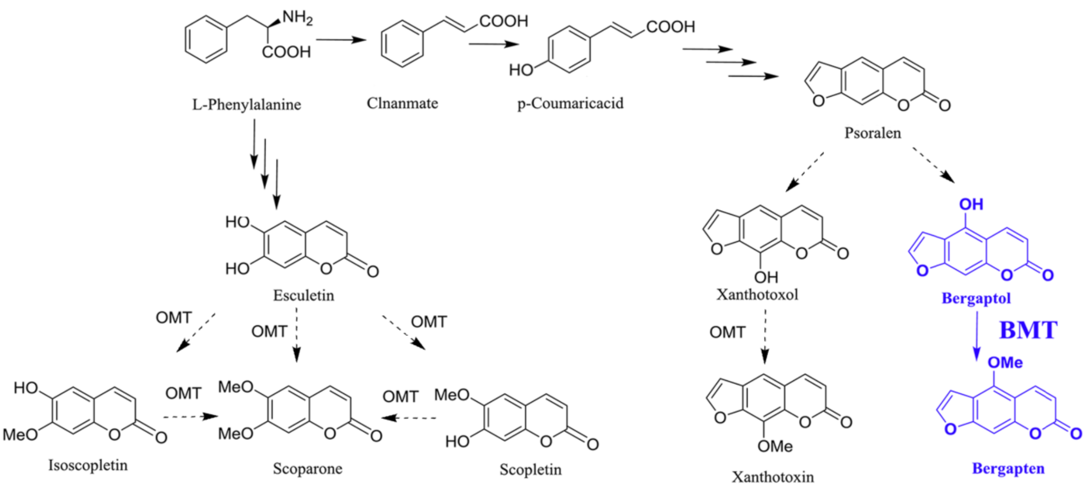

Figure 1. Schematic outline of OMTs involved in coumarin biosynthesis. OMT, *O*-methyltransferase; BMT, bergaptol *O*-methyltransferase. Blue solid line is BMT that had been functionally identified, while OMTs with dashed arrow are enzymes that had not been identified so far.

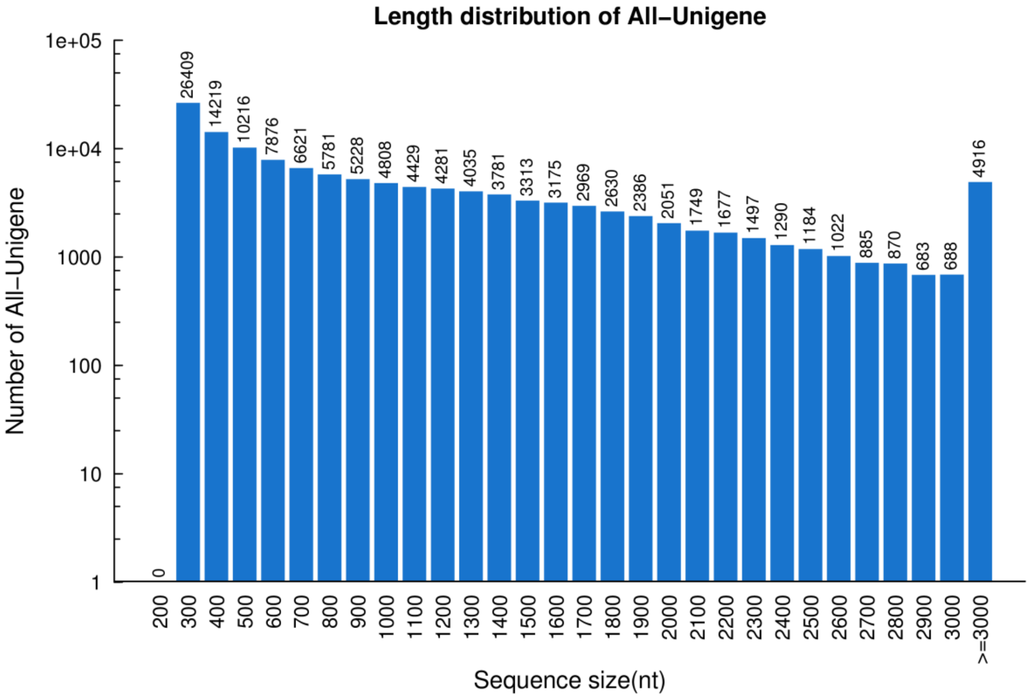

Figure S2. The length distribution of unigenes. The horizontal coordinates are unigenes' length and the vertical coordinates are numbers of unigenes.

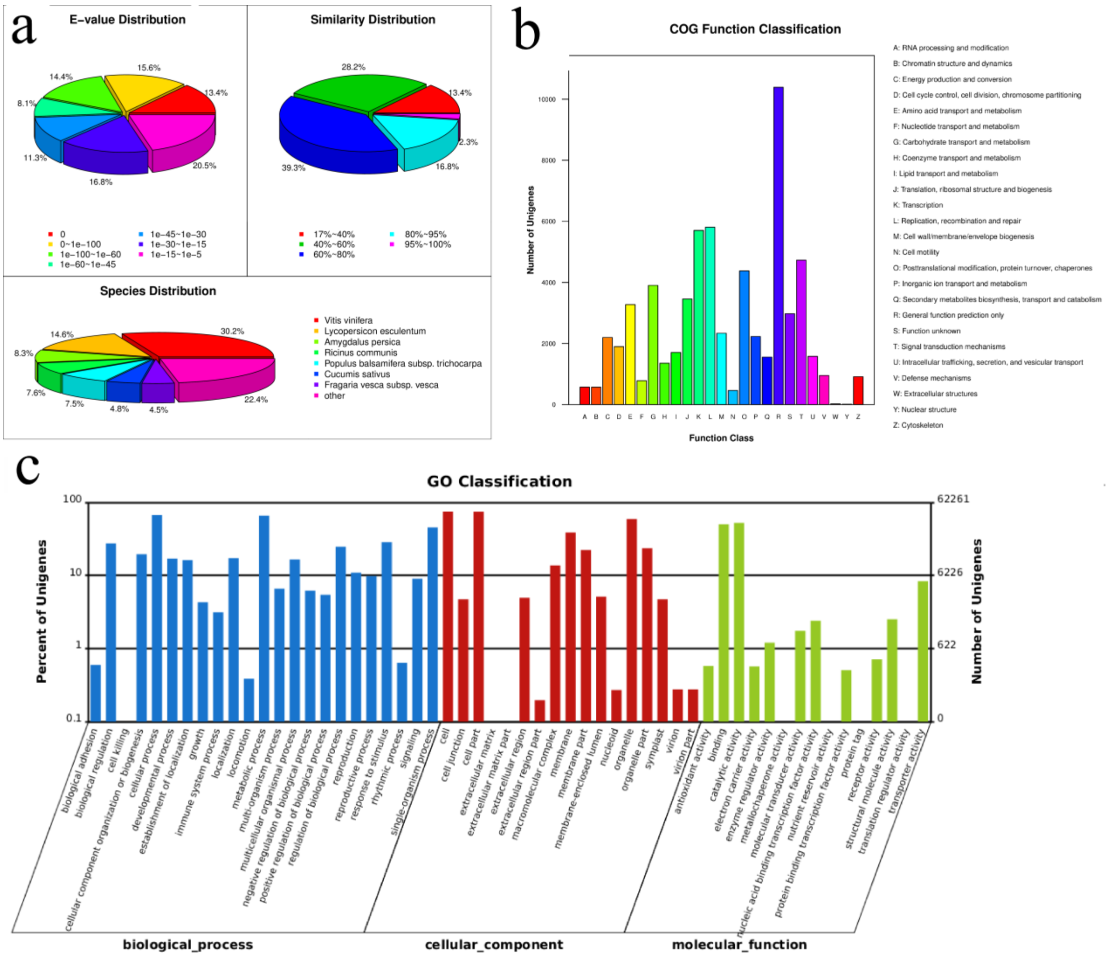

Figure S3. Functional annotation of unigenes with public database. (**a**) Class statistics, (**b**) COG function classification and (**c**) GO classification.

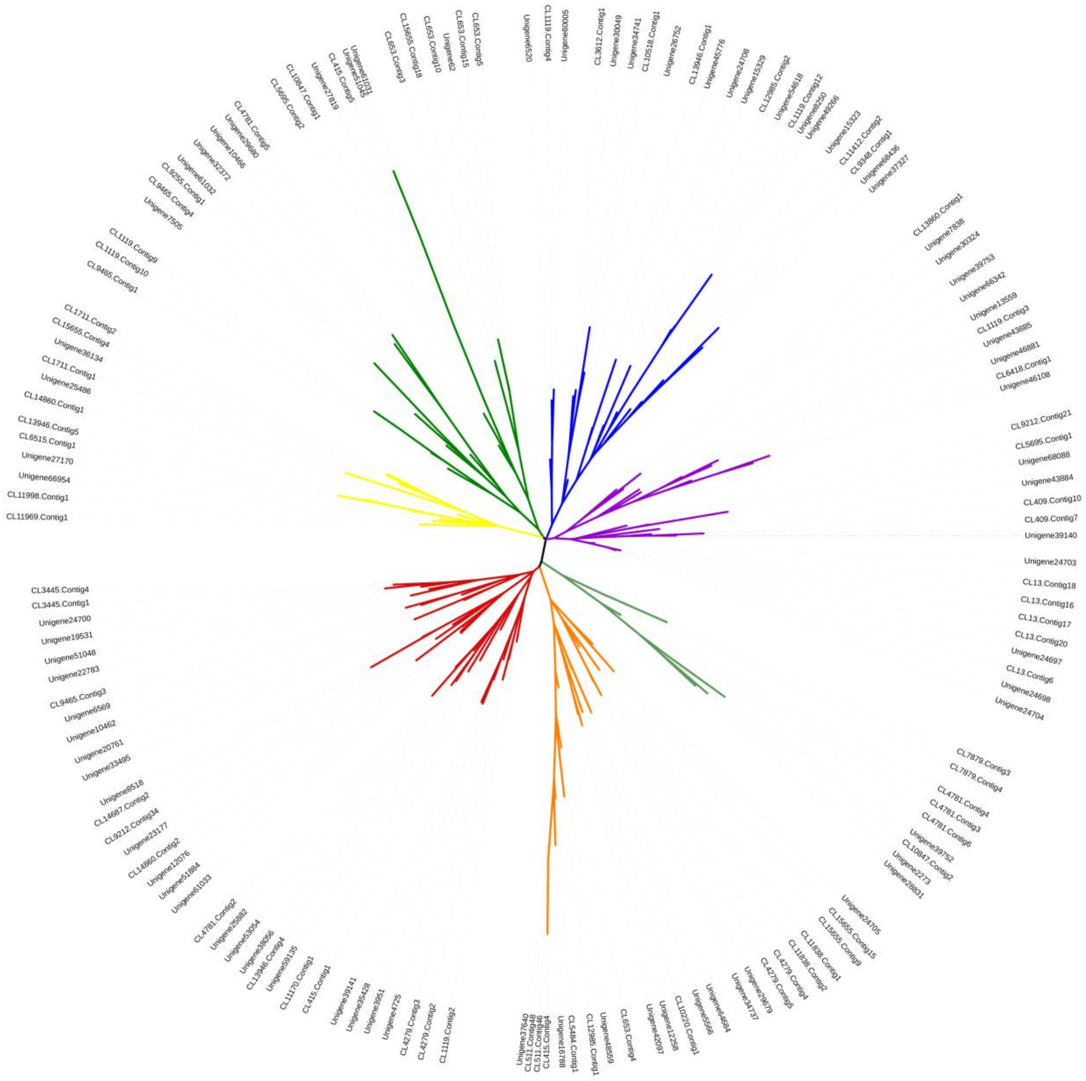

Figure S4. A simplified maximum-likelihood (ML) phylogenetic tree of 151 non-redundancy OMTs in *P. praeruptorum* transcriptome. Different color indicates the different kinds of OMTs.

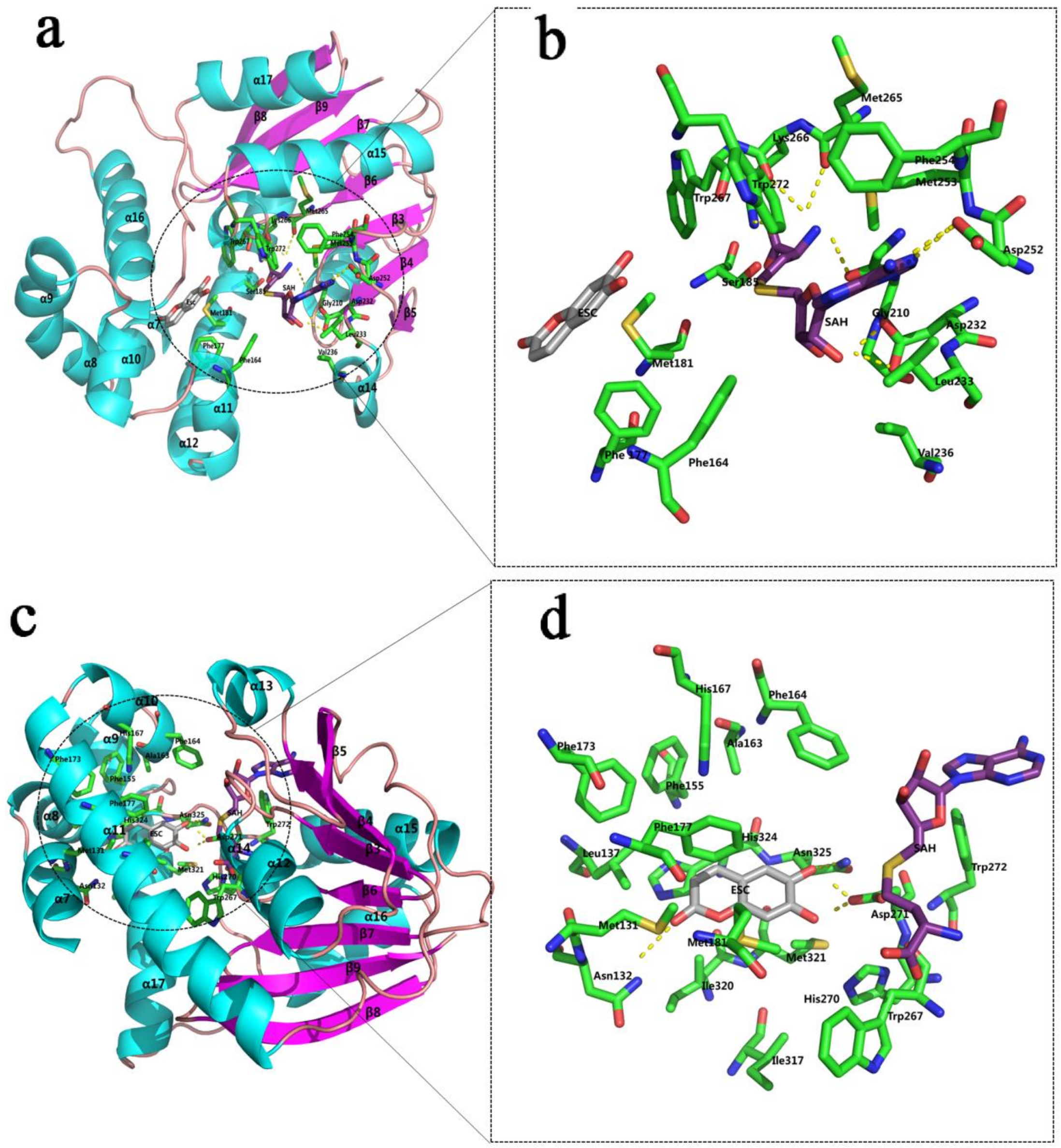

Figure S5. The docked binding sites of COMT-S. The ribbon diagram of the COMT-S in complex with SAH (**a**) and esculetin (ESC, **c**) and its local enlarged images (**b**, **d**). The secondary structure (both α-helix and β-sheet) and the important amino acid residues molecules contributing an atom within 4Å of SAH and ESC are shown in green sticks. SAH and ESC showed in violet purple and light blue sticks, respectively. The yellow dotted line is the hydrogen bond. The images were generated using the PyMOL Molecular Graphics System (version 1.7.4).

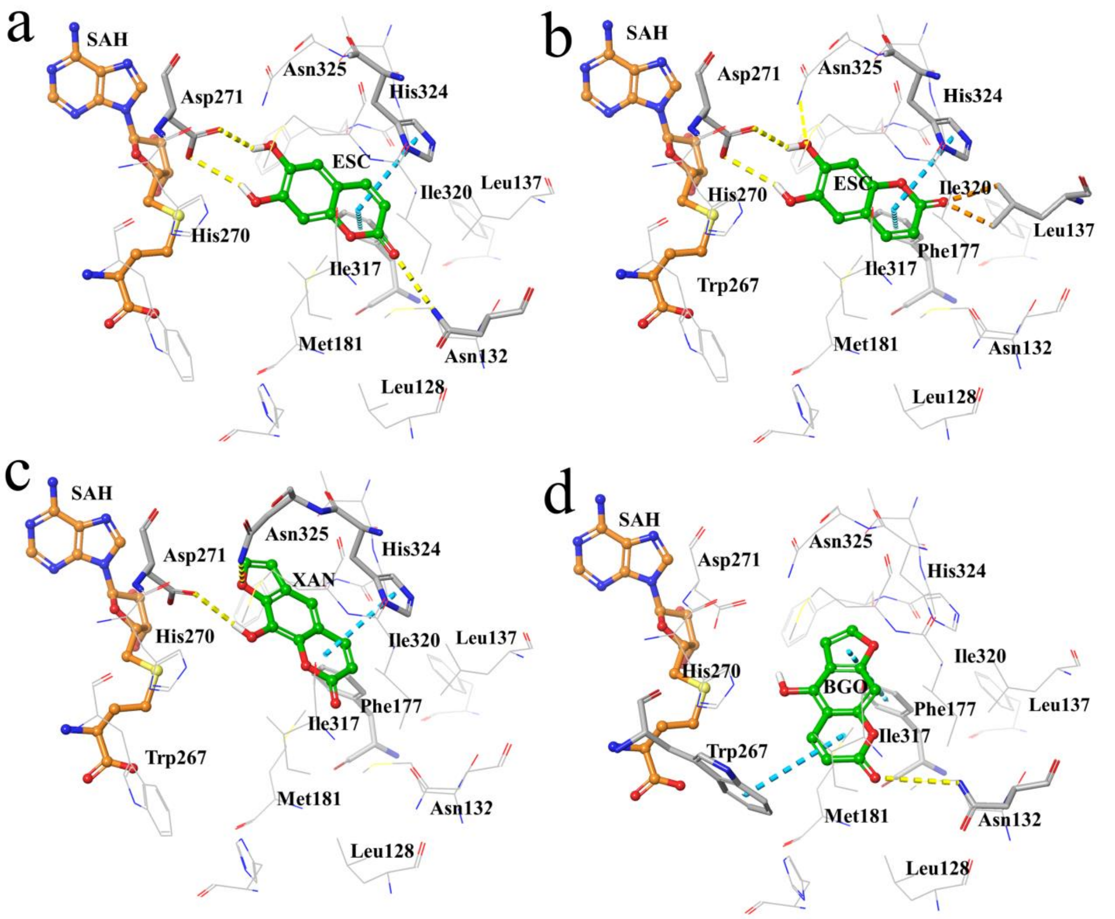

Figure S6. Docking of COMT-S with esculetin (**a**), xanthotoxol (**b**), bergaptol (**c**) and esculetin make a 180-degree reversal (**d**). The C atom of different coumarins is in green and the O atom in red. The yellow dotted line is the hydrogen bond, and the wathet dotted line is the π-π interaction.

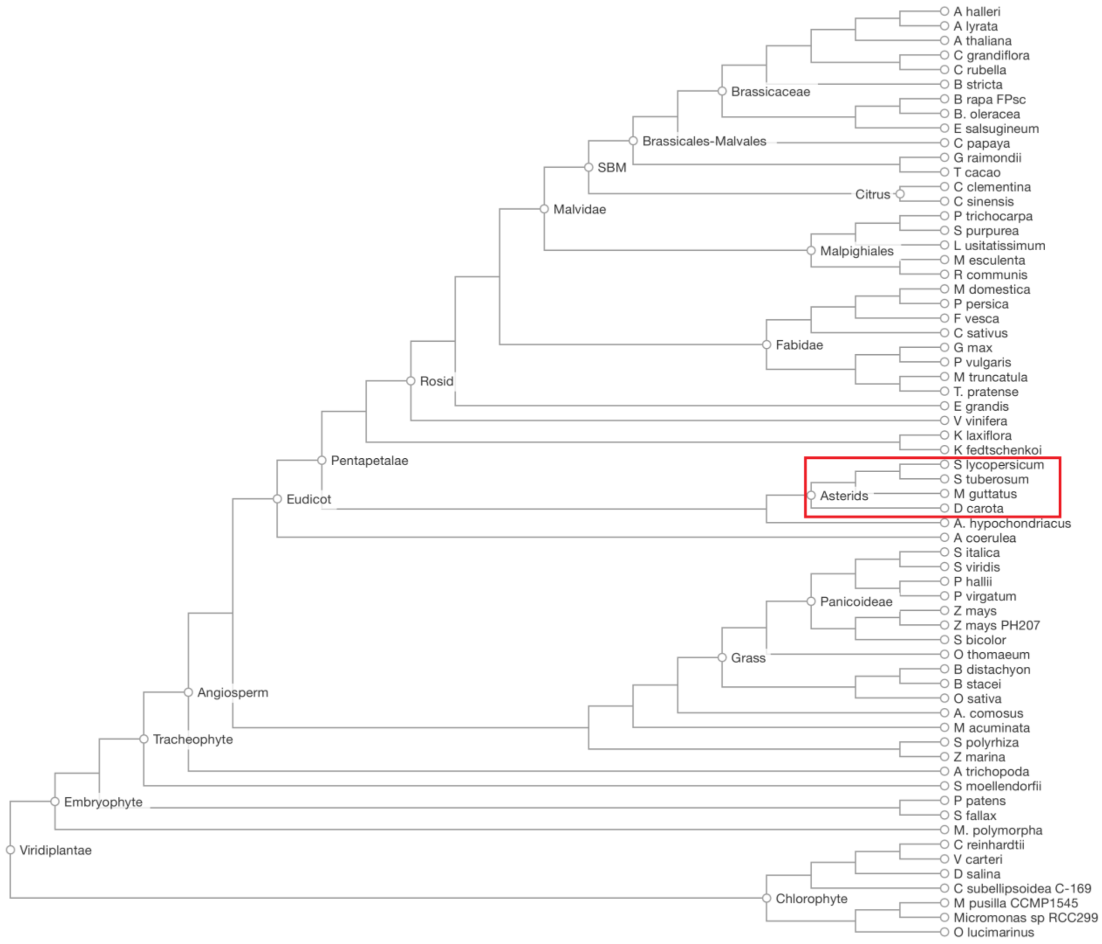

Figure S7. The phylogeny evolution of plant species with genome sequenced. *D. carota* in Asterids, which is a neighbor of *P. praeruptorum*, are marked with a red box. The tree is available at Phytozome (https://phytozome.jgi.doe.gov), which was shown at the right side at the main page in a green marked "tree representation".

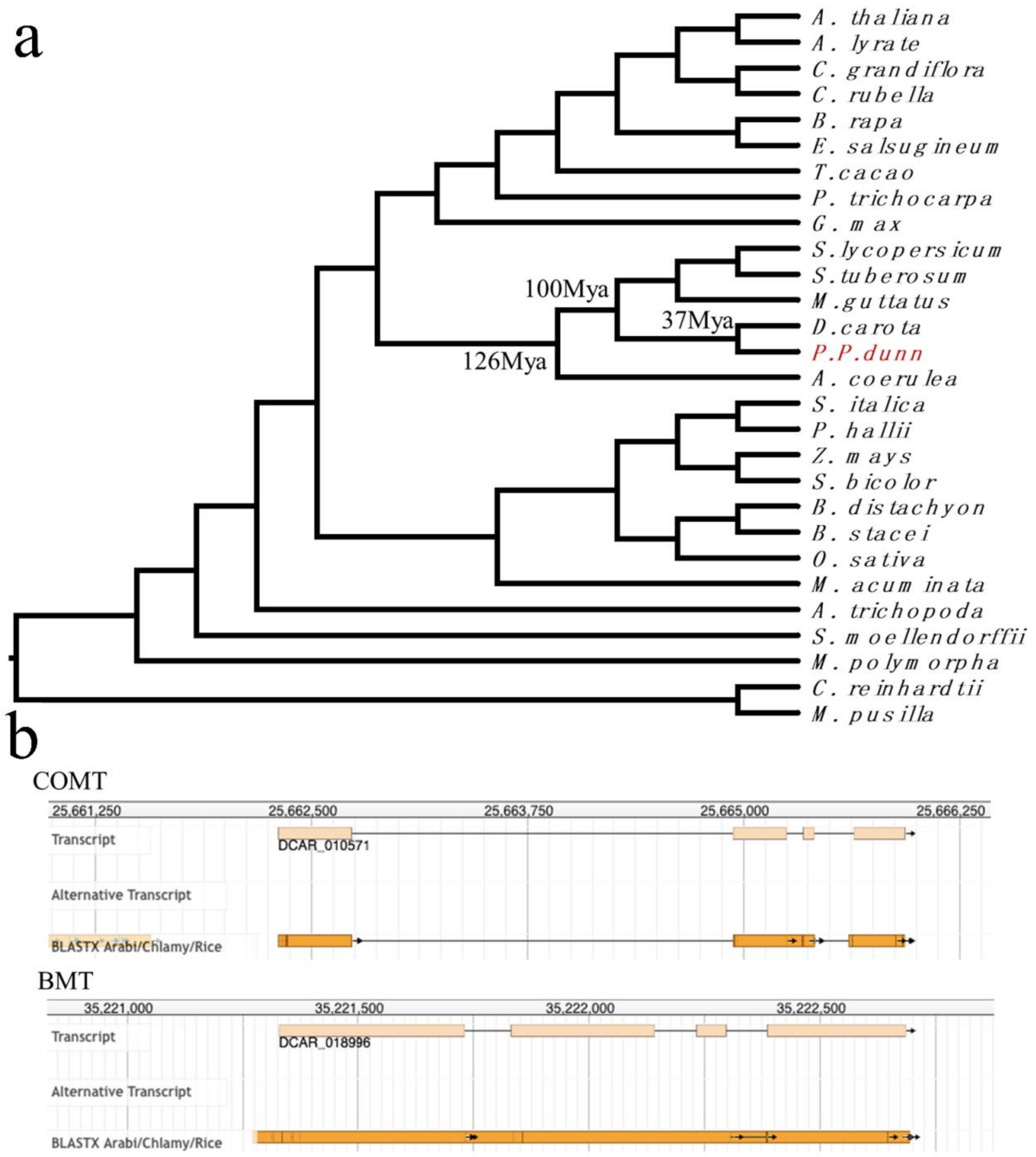

Figure S8. Evolutionary tree of representative species in Viridisplantae (**a**) and the genomic structure of BMT and COMT in *D. carota* (**b**). The divergent time of each representative species can be searched in the website: http://timetree.org.

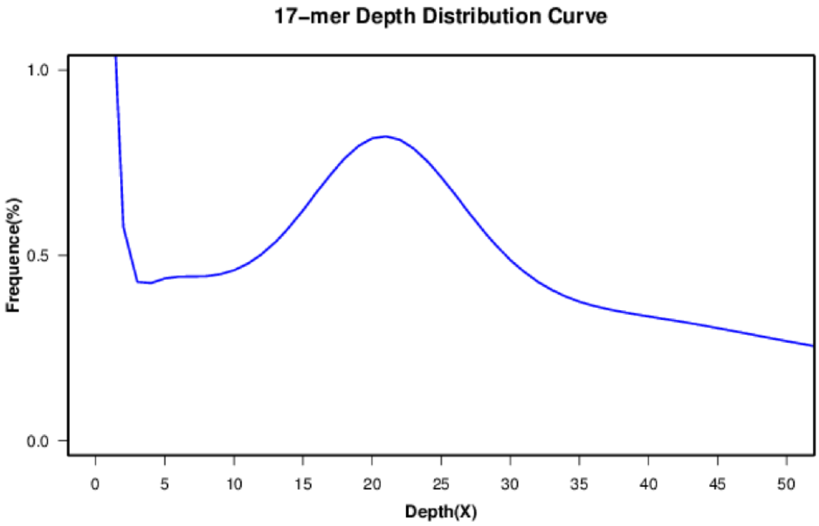

Sample	kmer	kmer_num	kmer_depth	genome_size(bp)
Pp-1	17	38,723,344,619	21	1,843,968,791

Figure S9. Seventeen-mer analysis and genome size estimation. The distribution curve shows a single peak distribution near 21, which is corresponding the kmer- depth. The genome size is the ratio of kmer-number to kmer- depth.

Supplementary Tables

Table S1. Output statistics of sequencing.

Samples	Total Raw Reads	Total Clean Reads	Total Clean Nucleotides (nt)	Q20 percentage	N percentage	GC percentage
WC-R	49,313,504	48,017,392	7,202,608,800	97.21%	0.01%	43.13%
WC-S	49,537,254	48,348,964	7,252,344,600	96.40%	0.01%	43.45%
WC-L	47,264,946	46,156,580	6,923,487,000	96.76%	0.01%	43.47%
WT-R	47,898,140	46,669,870	7,000,480,500	97.06%	0.01%	43.65%

Table S3. Data collection and refinement statistics of PpBMT, PpBMT complex, and PpCOMT-S.

	**PpCOMT-S**
	**6IWT**
**Data collection**	
Space group	C121
Unit cell	207.37
	70.39,59.65
	90.0,97.87,90.00
Resolution (Å)	2.53
Rmerge	0.048(0.586)*
I / σI	14.9(2.2)*
Completeness (%)	96.2(97.6)*
Redundancy	3.3(3.5)*
**Refinement**	
Resolution (Å)	66.58-2.53
No.of reflections	27,488
Rwork / Rfree (%)	18.9/26.1
No. of atoms	
Protein	5304
Water	4
Ligand	14
Avg.B-factor	82.00
R.m.s. deviations	
Bond lengths (Å)	0.008
Bond angles (°)	1.071
Ramanchandran plot	
Favored region	97
Allowed region	3
Outlier region	0

*Highest resolution shell is shown in parenthesis.

Table S4. Primers used in this study.

Primer Name	(5' to 3')
BMT-F	GGATCCTCTCAAGACGAAGAAGCTTGTGTGCTA
BMT-R	GCGGCCGCAAGCTTGTCGAGTTACTTGGAAAAT
COMT-F	GTGCCGCGCGGCAGCCATATGATGACTACAACTGAGCTAATC
COMT-R	TTGTCGACGGAGCTCGAATTCCTATTTAAGAAATTCCATAA
N132A-F	CCACTTCTGCTCATGGCCCAAGATAAGATCCTT
N132A-R	AAGGATCTTATCTTGGGCCATGAGCAGAAGTGG
L137A-F	AACCAAGATAAGATCGCTATGGAAAGCTGGTAC
L137A-R	GTACCAGCTTTCCATAGCGATCTTATCTTGGTT
F164A-F	TATGGAATGACAGCAGCTGAGTACCATGGAAAAG
F164A-R	CTTTTCCATGGTACTCAGCTGCTGTCATTCCATA
P171A-F	GAGTACCATGGAAAAGACGCCAGATTCAACAAAGTC
P171A-R	GACTTTGTTGAATCTGGCGTCTTTTCCATGGTACTC
M181A-F	GTCTTCAACCTGGGAGCGTCTAACCATTCCACT
M181A-R	AGTGGAATGGTTAGACGCTCCCAGGTTGAAGAC
S185A-F	GGAATGTCTAACCATGCCACTATTACTATGAAG
S185A-R	CTTCATAGTAATAGTGGCATGGTTAGACATTCC
D232A-F	AAGGGATTAACTTTGCTCTACCCCATGTTGTG
D232A-R	CACAACATGGGGTAGAGCAAAGTTAATCCCTT
L233A-F	GGGATTAACTTTGATGCACCCCATGTTGTGGAAG
L233A-R	CTTCCACAACATGGGGTGCATCAAAGTTAATCCC
D252A-F	GGAGCACGTTGGAGGTGCCATGTTTGTTAGCGTAC
D252A-R	GTACGCTAACAAACATGGCACCTCCAACGTGCTCC
M253A-F	CACGTTGGAGGTGACGCGTTTGTTAGCGTACCG
M253A-R	CGGTACGCTAACAAACGCGTCACCTCCAACGTG
K266A-F	GATGCTATTTTTATGGCGTGGATATGTCACGATTG
K266A-R	CAATCGTGACATATCCACGCCATAAAAATAGCATC
H270A-F	TATTTTTATGAAGTGGATATGTGCCGATTGGAGCGATGC
H270A-R	GCATCGCTCCAATCGGCACATATCCACTTCATAAAAATA
D271A-F	ATGAAGTGGATATGTCACGCTTGGAGCGATGCACATTG
D271A-R	CAATGTGCATCGCTCCAAGCGTGACATATCCACTTCAT
I317A-F	AAGAATGTCATTCATGCTGATGTCATAATGTTG

I317A-R	CAACATTATGACATCAGCATGAATGACATTCTT
I320A-F	ATTCATATTGATGTCGCAATGTTGGCACATAAT
I320A-R	ATTATGTGCCAACATTGCGACATCAATATGAAT
M321A-F	CATATTGATGTCATAGCGTTGGCACATAATCCC
M321A-R	GGGATTATGTGCCAACGCTATGACATCAATATG
N325A-F	ATAATGTTGGCACATGCTCCCGGTGGAAAAGA
N325A-R	TCTTTTCCACCGGGAGCATGTGCCAACATTAT
